# Impact of implicit vs. explicit Instructions on tactical performance in under-20 soccer players

**DOI:** 10.3389/fspor.2024.1441125

**Published:** 2024-09-09

**Authors:** Cristóvão de Oliveira Abreu, Layla Maria Campos Aburachid, Igor Mendes Lima, Felipe A. Moura, Bruno L. S. Bedo, Schelyne Ribas, Gibson Moreira Praça

**Affiliations:** ^1^ CECA/UFMG Soccer Science Center, Sports Department, Universidade Federal de Minas Gerais, Belo Horizonte, Brazil; ^2^ GEMEPE/UFMT, Theory and Foundation in Physical Education Department, Universidade Federal de Mato Grosso-UFMT, Cuiabá, Brazil; ^3^ GEMEPE/UFMT, Physical Education Department, Universidade Federal de Mato Grosso-UFMT, Cuiabá, Brazil; ^4^ Sport Sciences Department, State University of Londrina, Londrina, Brazil; ^5^ Sport Department, School of Physical Education and Sports, University of São Paulo, São Paulo, Brazil

**Keywords:** small-sided games, informational constraints, ecological dynamics, tactical training, position data

## Abstract

**Introduction:**

This study aimed to verify whether implicit and explicit informational constraints generate differences in tactical performance and behavior in U-20 national-level soccer players.

**Methods:**

Thirty-two under-20 male athletes from two clubs participated. Four 4-a-side small-sided games (SSG) protocols were used: R1 - explicit rule for high-press marking, R2 - implicit rule for high-press marking, R3 - game with both previous rules simultaneous and FR - free game, without additional rules. SSGs comprised 4 vs. 4+ goalkeepers games on a 42 m × 29 m field. Position data from 10 Hz Global Positioning System (GPS) devices were used to evaluate individual and collective tactical behavior (spatial occupation metrics) and performance (interpersonal coordination). MANOVA was used for normally distributed variables, and Friedman's with Dunn or Bonferroni *post hoc* was used for variables without normal distribution. For SEI, an ANOVA was used with Bonferroni *post hoc*.

**Results:**

The R1 protocol showed higher SEI, length, width, and LpWratio than the FR protocol (*p* = 0.009). There was an effect of the different protocols in SEI values (*p* < 0.001). Under the explicit rule, players also showed the highest in-phase interpersonal coordination values (*p* < 0.001).

**Discussion:**

Providing players with explicit tactical instructions improves tactical performance acutely for high-pressing defensive actions.

## 1 Introduction

Small-sided games (SSGs) are adopted in football training to simultaneously develop physical, physiological, technical, and tactical skills (1). Moreover, SSGs allow for keeping the internal logic of the sport (increasing the specificity of the training) while providing coaches with the possibility to manipulate constraints to adapt the task difficulty to players’ levels and sessions' aims (2). When manipulating constraints, the players' exploratory behavior can be shaped intentionally by the coach to focus on specific affordances within the available landscape (3, 4). Within the constraints' manipulation landscape, informational constraints refer to the information available—or made available—for action (5). Coaches usually change informational constraints by providing players with additional information about the task. This information can be provided explicitly—with specific and clear goals—or implicitly—favoring exploration throughout the task. The literature has extensively explored task constraints, such as pitch size (6, 7) and the number of players (8, 9). However, the impact of informational constraints remains under-researched, especially in team sports such as soccer.

Although scarcely investigated, informational constraints have previously been shown to shape players' behaviors in game-based tasks, such as SSGs. For example, a study showed that when players were informed about a shorter bout duration, they increased their pace despite the total game time being similar across the conditions (10). Another study showed that a strong feedback strategy decreased players' tactical performance compared to an unobtrusive feedback strategy (11). Therefore, it is arguable that providing players with instructions can direct their attention towards specific affordances. However, the available literature on informational constraints and small-sided games focused on general information, including match status (12), bout duration (10), and feedback (11). Little is known about the impact of explicit tactical instructions on players' behaviors during SSGs.

Raab (13) suggests that adopting implicit or explicit learning strategies should rely on task complexity. At this point, Lola et al. (14) observed that in highly complex situations, the explicit learning group outperformed the implicit group in a badminton task. The authors suggested that the verbal instructions guided explicit learning and helped novices focus on the most relevant environmental cues, improving their learning (13). Raab (13) points out that decisions under high-complexity contexts require some explicit learning to quickly improve. According to the author, “if-then” explicit rules (e.g., “*you will score a point if you regain ball possession in the offensive midfield*”) can facilitate decision-making during the task. However, the complexity of this task can increase when the defending team coordinately advances on the pitch to constrain the space and time of the attacking team (known as high-press defending). This complexity increase could be explained by the increased information available in the task (13) and a restricted time to accept one of the affordances within the available landscape. At this point, it could be assumed that explicit learning might be necessary when perceptual-cognitive demands are high (13), which seems to be the case when dealing with elite team sports, such as soccer. However, to our knowledge, no studies are available investigating the possible effect of implicit and explicit informational constraints on tactical performance during ecologically-representative complex tasks in soccer (such as SSGs).

The literature suggests that some tactical contents are more complex for players to learn and execute (15, 16). For example, it has been shown that the performance in defensive coverage actions is higher than in defensive concentration actions (17). Interestingly, while effective defensive coverages require coordinating efforts among two teammates, effectively concentrating (protecting the central area of the pitch) might require the whole team to coordinate, which might explain the higher complexity of this principle. Reinforcing this rationale, the number of players seems to affect the players' decisions (18, 19). Caso and Kamp (18) showed that games with fewer players generated more creative decisions than larger formats. Also, Silva et al. (16) demonstrated that the space management of the players was better in the smaller format (3 vs. 3) than in the larger one (6 vs. 6). Therefore, the more players involved in an action, the more complex it is expected to be.

Based on the abovementioned rationale, the high-press defensive strategy can be assumed to be a highly complex tactical content due to the need for coordination among teammates for better execution (20). The high press is characterized by trying to regain the ball as close to the opposing goal as possible. This defensive strategy has been linked with success in elite soccer as more advanced defensive pressure allows ball recoveries in more advantageous pitch positions (21, 22). González-Rodenas, Calabuig, and Aranda (23) suggested that the closer to the opponent's goal the ball is recovered, the higher the possibility of scoring a goal and creating goal-scoring opportunities. Hughes and Lovell (24) showed that 49.45% of the balls recovered in the opponent's defensive midfield led to shots on goal, and 7.69% of the actions generated goals. Therefore, stimulating the players to high-press the opposing team is relevant for defensive success in soccer. When practicing this content, implicit or explicit informational constraints can be manipulated. Therefore, training this content seems a promising context for testing whether implicit or explicit informational constraints influence players' tactical performance in soccer. For example, scoring rules could be explicitly (i.e., giving a point score to the defending team every time they recover the ball in their offensive midfield) or implicitly (giving a point score to the offensive team every time they can shoot on goal from their offensive midfield) manipulated. These task constraints will shed light on how informational constraints affect players' actions during SSGs.

Studies have used different Electronic Performance and Tracking Systems (ETPS), such as Global Positioning Systems (GPS) position data, to evaluate players' positioning in game-based tasks, including SSGs (25–27). Conventional game analysis approaches rely on discrete event-based data, while innovative methods suggest integrating position-based data to comprehensively understand the game's inherent dynamics, often beyond traditional analysis's scope (28, 29). Position-based data enable the analysis of the actions executed by players as they adapt to dynamically changing environments (30), considering both individual and collective perspectives (31). Studies have adopted measures of movement coordination to quantify performance through space-time interactions among players (32). Classical studies relied on players' and teams' spatial occupation metrics, such as length, width, and spatial exploration, to describe space management during game-based tasks and official matches (33–35). Regarding movements on the field in length and width (i.e., in the longitudinal and lateral axes, respectively), it was shown a reduction in the percentage of time that dyads (i.e., all possible pairs of players from the same team) maintained coordination in the in-phase pattern in SSGs that involved modifications to the spatial references of the playing field when compared to SSGs that did not undergo these modifications (36, 37). Also, another study observed that in situations of defeat, the team showed lower coordination percentages, indicating it to be a characteristic that distinguishes winning from losing teams (38). As the high-press defensive strategy is linked to the pitch's occupation, evaluating game-based tasks oriented to this pedagogical purpose using position-based data is theoretically founded.

Although the literature on the Constraints-led Approach (CLA) using SSGs has advanced remarkably over recent years, the influence of explicit and implicit informational constraints on players' actions remains unknown. Investigating this would improve the understanding of players' adaptive actions throughout game-based tasks, as coaches regularly provide information during training. Therefore, this study aimed to compare the tactical behaviour and performance during SSGs with explicit and implicit informational constraints. The literature suggests that the benefits of explicit learning are higher under high-complexity contexts (13). Therefore, the explicit rule condition is expected to lead to a higher tactical performance, demonstrated by a higher percentage of in-phase coordination tendency (39).

## 2 Methods

### 2.1 Sample size estimation

The sample was statistically determined after a pilot study conducted over four training sessions of a U-17 male soccer team. The pilot study comprised a specific data collection session in which players engaged in small-sided games similar to those adopted in the main data collection. For this pilot study, the technical staff classified the participants as defenders, midfielders, and forwards. All the players were divided into three 4-a-side teams composed of one defender, two midfielders, and one forward. Players performed small-sided games with the same rules adopted in the main study (see the section “procedures”) for four minutes, engaging in all four experimental conditions during the four data collection sessions. Finally, the tactical performance obtained through the System of Tactical Assessment in Soccer (FUT-SAT (40); was used as a criterion for sample size estimation. The sample size calculation was performed using the software G*Power (Version 3.1.9, Universitat Kiel, Germany) considering the following parameters: *f* tests family, ANOVA: repeated measures within factors, effect size *f* = 0.219, partial *η*^2^ = 0.046, number of groups = 1, number of measurements = 4, alfa = 0.05, beta = 0.80, following the literature recommendation (41). The effect size adopted in the sample size estimation was obtained from the previous pilot study. The sample size estimation indicated a minimum of twenty observations to ensure the expected effect size.

### 2.2 Participants

The final sample comprised 32 U-20 male soccer athletes (18.2 ± 1.2 years; 9.2 ± 1.6 years of practice in the sport and 5.1 ± 0.9 years of experience in official competitions) from two national-level soccer academies. The final sample was higher than originally determined to account for sample dropouts and injuries and included players from different clubs, increasing the sample's representativeness. Inclusion criteria comprised belonging to officially registered clubs, competing in U-20 regional-to-national level competitions, and engaging in at least five weekly training sessions. Participants were excluded if they presented any injuries at the beginning of the data collection or could not participate in all data collection sessions. Participants are classified as tier 3 according to the literature (42). The players' level was chosen due to the complexity level of the tactical content analyzed in the current study, which is expectedly highly complex. Also, only male players were included, as the availability of competitive youth academies in the region was limited at the time of the data collection. The local ethics committee approved the study (Approval No. 46940821.7.0000.5149), and the principles of the Declaration of Helsinki were followed. Written informed consent was obtained from all participants and their legal guardians, ensuring their understanding and voluntary participation in the research.

### 2.3 Procedures

#### 2.3.1 Team composition

Previous studies showed the influence of playing position and tactical knowledge on players' behaviors (43). Therefore, both criteria were adopted to ensure that balanced teams would be composed, similar to previous studies on this topic (44, 45). The processes for organizing balanced teams, described below, lasted two days in each club.

**Figure 1:**
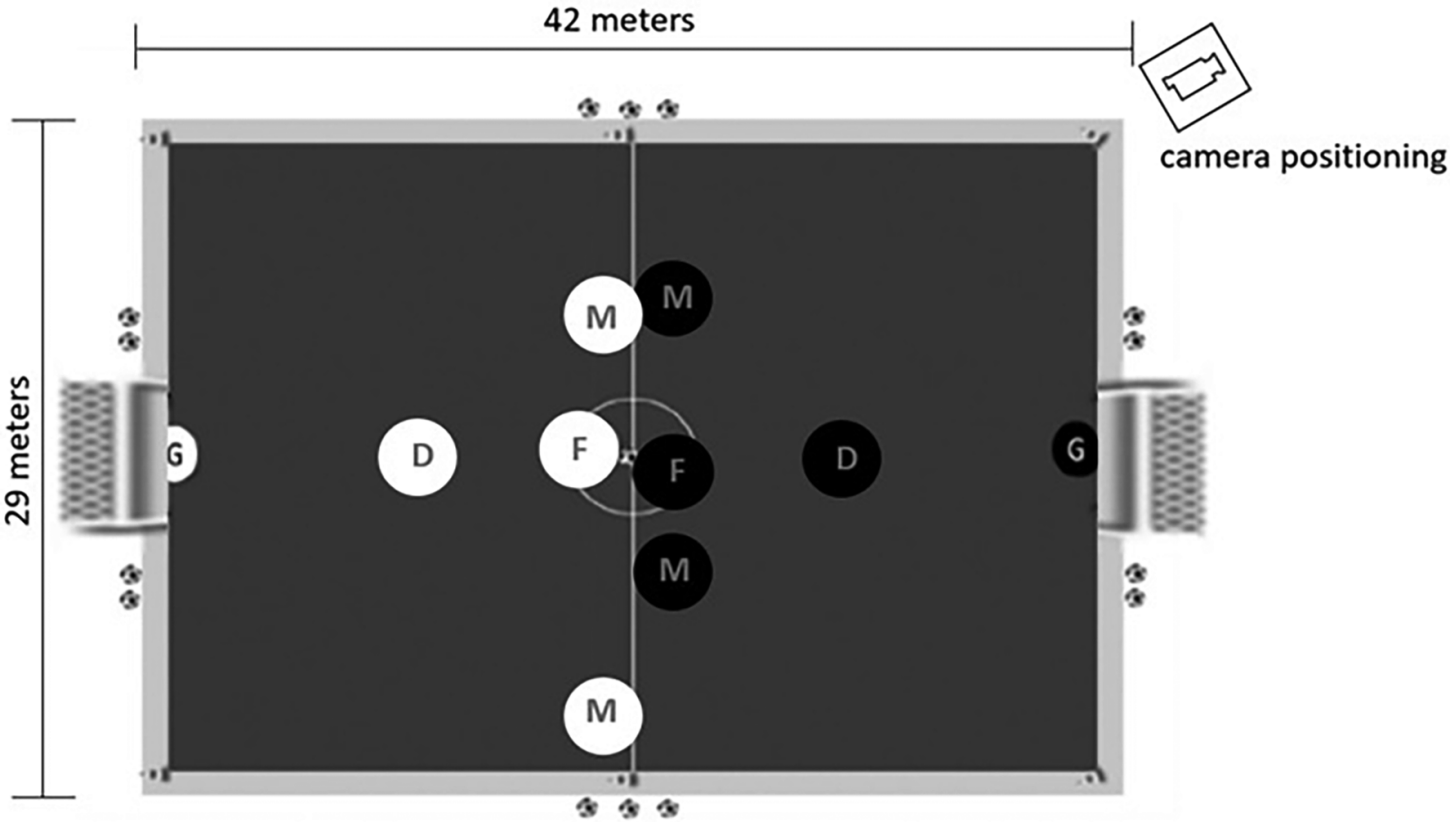
Representation of the procedures of data collection on each club.

To conduct the team composition procedures, the technical staff classified the participants within each club as defenders, midfielders, and forwards. These groups performed the field test of the System of Tactical Assessment in Soccer (40) adopted to rank the players within each playing position. This classification was based on the percentage of successful tactical actions, as previously suggested in the literature (46). The two best defenders and forwards and the four best midfielders were used to compose teams A and B, which played only against each other, while the remaining participants composed teams C and D (see Figure 1), which also played only against each other. Keeping teams and matches stable during the data collection was required as previous studies showed the players' tactical level (47) and the level of the opposition (48) could influence the players' behavior. The same experimental design was repeated in the second team that participated in the study (*n* = 32).

#### 2.3.2 Data collection procedures

This study was a randomized, within-subject trial where the participants engaged in four experimental conditions on different days. Participants could not be blinded as the informational constraint manipulated involved providing them with specific action rules. The randomization was conducted to allow all experimental conditions to be played in all possible orders within a session (from bout one to bout four), reducing the influence of the learning effect on the observed responses (49).

After the teams were formed, a familiarization session was held in which players participated in each experimental condition for four minutes. In this session, players engaged in all the formats they would play during the main collection. Also, they were allowed to ask questions regarding the rules and scoring systems, ensuring comprehension of the study's design. Players were considered familiar with the experimental conditions’ when no remaining questions were raised.

In sequence, the main data collection started. Each data collection session started with a 10-min standardized warm-up composed of on and off-the-ball displacements with changes in direction and speed. After the warm-up, in each session, participants played three SSGs from the same experimental condition for four minutes, with four minutes of passive recovery (1:1 work-to-rest ratio). This procedure was adopted to reduce the influence of fatigue on players' performance during the SSGs and was repeated for five data collection sessions in each club for two weeks. The experimental conditions' presentation order was randomized and balanced to control for learning effects. The data collection sessions started at the same time every day to control for the influence of the circadian rhythm on players' responses (50). Four experimental conditions were investigated: FR (free-rule), R1 (rule 1 - explicit rule), R2 (rule 2 - implicit rule), and R3 (combination of rules 1 and 2). The FR contidion is a free-play game without any additional restrictions. In the R1 condition, an explicit rule for high-defensive pressure was applied. In this, both teams were told they would score two additional points every time they regained the ball in their offensive midfield, stimulating them to move forward during the defensive phase. Recovering the ball in the offensive midfield will likely increase the possibility of shooting on goal and scoring goals (23). The R2 condition involved an implicit rule for high-defensive pressure that incentivized players to retrieve the ball from the opposing team's pitch without being explicitly told to do so. Specifically, teams would receive two additional points each time they could shoot on goal from their offensive midfield. While the defending team was not given any specific instructions, they may have implicitly learned that implementing a high-press defending strategy would be the most effective way to prevent the other team from scoring. Finally, in the R3 conditions, players were given the instructions mentioned above. Providing encouragement or verbal instructions during SSGs was not permitted.

#### 2.3.3 Small-sided games

**Figure 2:**
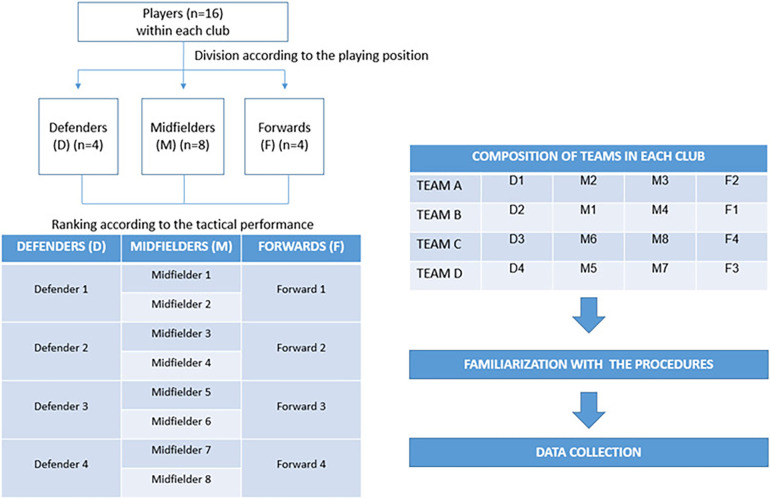
The standard format of the small-sided game adopted in the study.

After teams were composed, players engaged in the 4-a-side SSGs (Figure 2) in a 42 × 29 m pitch. This pitch has an area per player equal to 121.8 m^2^, similar to the 121.5 m^2^ observed in the 36 × 27 m pitch in the 3-a-side SSG extensively adopted in the literature (40, 47). The 4-a-side SSG was adopted as previous studies investigated similar dependent variables in this format (16, 19, 51), which facilitates the interpretation and discussion of the results. The SSGs were played on a natural surface grass pitch, and all official rules, including the offside, were applied. The adoption of the offside rule, which differs from other previous studies, intended to increase the representativeness of the task, as it has been shown that removing this rule increases in-depth players' spatial exploration (52). White elastic bands marked the sidelines, the midfield, and the goal lines to facilitate players' identification of the available space. The literature showed that this strategy to mark the pitch facilitates players' synchronization during SSGs (53). Extra balls were positioned at the sidelines to facilitate the restart of the game when the ball was kicked out of the field. Goalkeepers were part of the study but were removed from the statistical and positional analyses to avoid misinterpretations.

### 2.4 Instruments

#### 2.4.1 System of tactical assessment in soccer—FUT-SAT

The tactical performance of the players, used as a criterion for team composition, was evaluated using the System of Tactical Assessment in Soccer—FUT-SAT (40). The assessment considers ten core tactical principles: five related to the offensive phase (penetration, offensive coverage, depth and width, mobility, and offensive unity) and five related to the defensive phase (delay, defensive coverage, balance, concentration, and defensive unity). The analysis was performed by an expert in the system who evaluated players' actions from the video footage. The camera was positioned high to allow all players to be captured over the whole bout. In addition, the software *Soccer Analyser®*, allowed the pitch to be split into twelve game zones, facilitating the interpretation of the tactical principles. Considering the observational characteristics of the data provided by the FUT-SAT, a within- and between-observers agreement analysis was required to ensure the consistency and external validity of the observations. For this purpose, 10% of the videos were reassessed by the original expert and second expert analyst, following the literature guidelines (54). The Intraclass Correlation Coefficient (CCI_3,1_) was used to calculate the agreement. The values ranged within-observer 1 ICC = 0.936 [IC 95% = 0.718–0.987; F_(7,7)_ = 30.48; *p* < 0.01] and observer 2 ICC = 0.917 [IC 95% = 0.644–0.983; F_(7,7)_ = 23.04; *p* < 0.01]. The values for between-observer agreement ICC = 0.948 [IC 95% = 0.883–0.977; F_(23,23)_ = 37.26; *p* < 0.01]. The literature shows that these values are considered satisfactory (55). Every small-sided game was recorded (JVC® HD Everio model GZ-HD520 Camcorder, Brazil) for this assessment.

#### 2.4.2 Electronic performance and tracking systems (EPTS)

Electronic performance and tracking systems (EPTS) were adopted in the current study to track players' positions and displacements in the game-based tasks. The adopted EPTS was a 10 Hz GPS device with Bluetooth or Adaptative Network Topology (ANT+), adopting a GPS constellation system (EE. UU.) embedded with a triaxial accelerometer, gyroscope, and digital compass (Polar®, Team Pro, Kempele, Finland) and processed in MATLAB 2010a (The MathWorks Inc., Natick, MA, USA). The manufacturer does not provide information regarding satellite availability in each data collection. The equipment used in the current study has been previously tested in the literature for the accuracy and reliability of movement detection (56). Each player wore a chest strap with the device attached to it. Latitude and longitude data were downloaded using the manufacturer software (Polar Team Pro Web Service, Polar®, Team Pro, Kempele, Finland), synchronized, and converted into meters using the Universal Transverse Mercator (UTM) coordinate system and a MATLAB routine (57). The data were smoothed using a second-order 0.5 Hz Butterworth low pass filter. After converting the positional data into meters, a rotation matrix was calculated for each SSG with the field vertices positions, aligning the length of the playing field with the x-axis and the width with the y-axis. Then, the rotation matrix was applied to the athletes’ positional data for alignment with the playing field referential (20).

The following variables were calculated: (a) width, determined by the distance between the players most to the right and left of the team; (b) length, determined by the distance between players who are most vertically apart (26); (c) length by width ratio (LPWratio), which indicates the team's preferred positional axis, with higher values indicating a deeper positioning (48); (d) the centroid of each team, calculated as the average position of each player on each instant (58) and used to calculate the distance between the centroids, understood as the Euclidian distance between these points every instant; (e) spatial exploration index (SEI), defined as the average difference between a player's average position and his actual position at each moment of the game (26). The SEI indicates how a player explores the pitch, irrespective of the preferential axis, with higher values indicating a more exploratory behaviour (52). The width, length, LPW ratio, and stretch index were collectively measured, while the SEI was individually analyzed. Position data from this device was previously tested for inter and intra-session reliability and showed acceptable values (59).

Besides the linear measures, players' interpersonal coordination tendencies were evaluated for both longitudinal and lateral transformed coordinates using the vector coding technique (32). The coupling angle between two-time series represents an instantaneous spatial relationship that may be characterized by different coordination patterns, including anti-phase and in-phase coordination patterns (32). An in-phase pattern is considered when coupling angles are around 45° (from 22.5 to 67.5°) and around 225° (from 202.5 to 247.5°) (i.e., the positive diagonal). This happens, for example, when two players are moving forward to press the opposing player. On the other hand, an anti-phase pattern is considered when coupling angles are around 135° (from 112.5 to 157.5°) and around 315° (from 292.5 to 337.5°) (i.e., the negative diagonal). This happens, for example, when fullbacks move in opposite directions to increase the team's width.

### 2.5 Data analysis

Initially, data were screened using descriptive statistics. Next, the assumption of normality of the data was tested using Shapiro-Wilk's test. Then, a MANOVA with Bonferroni's *post hoc* was adopted to test the influence of the experimental condition on the tactical responses. This technique of including all dependent variables in the model was chosen to reduce the inflation of the type I error (60). When deviations from a normal distribution (distance between centroids, mobility, and depth and width) were found, Friedman's test with Dunn's *post hoc* was adopted. For the SEI, an ANOVA with Bonferroni's *post hoc* was adopted. The effect size (*η*^2^*p*) was classified into no effect (*η*^2^*p* < 0.04), minimum effect (0.04 ≤ *η*^2^*p* < 0.25), moderate effect (0.25 ≤ *η*^2^*p* < 0.64), and strong effect (*η*^2^*p* ≥ 0.64) (61). The level of significance was set at 5%. All analyses were conducted using the statistical software IBM SPSS Statistics (IBM Corp. Released 2010. IBM SPSS Statistics for Windows, Version 19.0. Armonk, NY: IBM Corp.).

Considering the observational characteristics of the data provided by the FUT-SAT, a within- and between-observers agreement analysis was required to ensure the consistency and external validity of the observations. For this purpose, 10% of the videos were reassessed by the original expert and second expert analyst, following the literature guidelines (54). The Intraclass Correlation Coefficient (CCI_3,1_) was used to calculate the agreement. The values ranged within-observer 1 ICC = 0.936 [IC 95% = 0.718–0.987; F_(7,7)_ = 30.48; *p* < 0.01] and observer 2 ICC = 0.917 [IC 95% = 0.644–0.983; F_(7,7)_ = 23.04; *p* < 0.01]. The values for between-observer agreement ICC = 0.948 [IC 95% = 0.883–0.977; F_(23,23)_ = 37.26; *p* < 0.01]. According to the literature, these values are considered satisfactory (55).

## 3 Results

**Figure 3:**
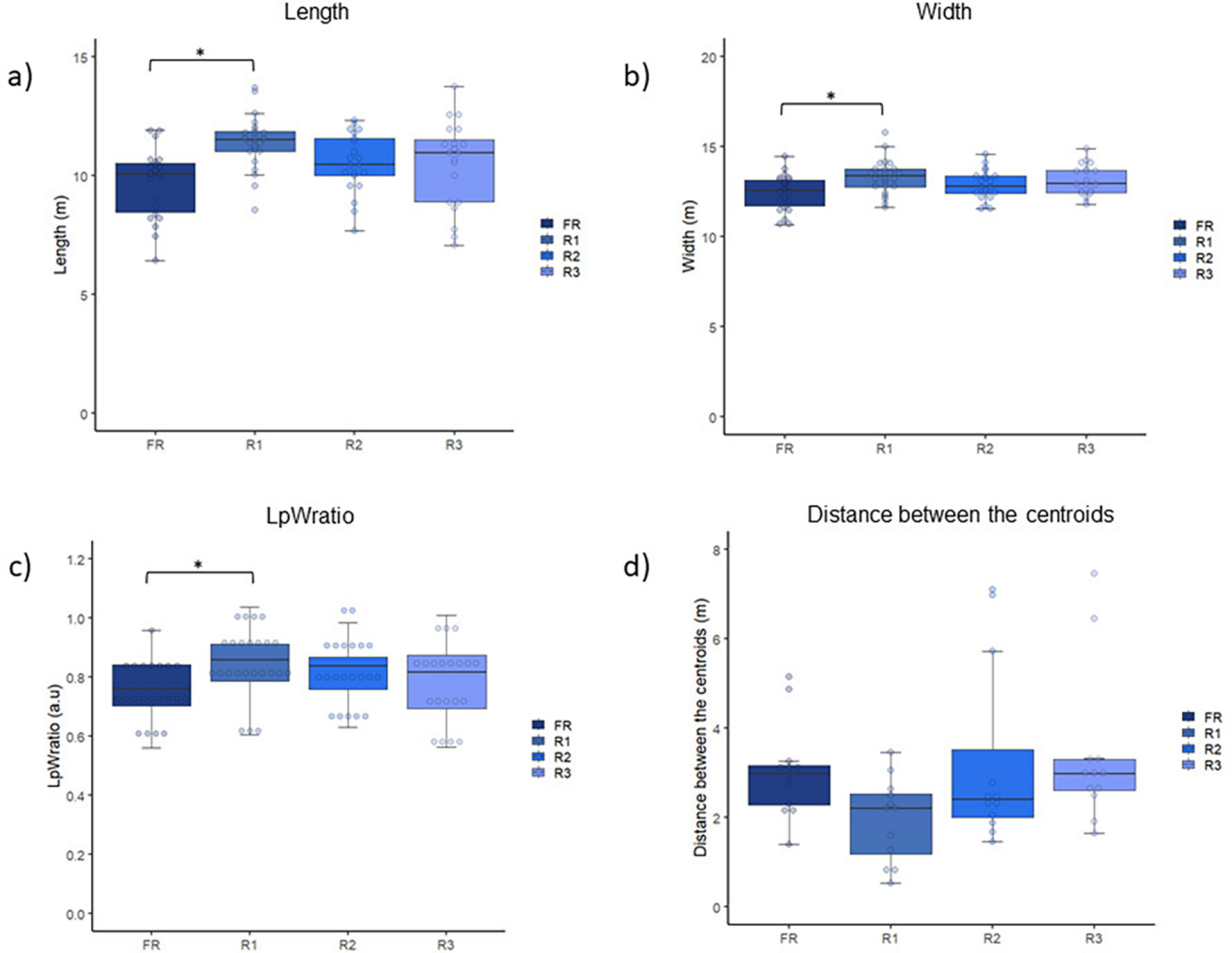
Comparison of the **(A)** length, **(B)** width, **(C)** lpWratio, and **(D)** distance between centroids between the four experimental conditions. *significant differences.

The MANOVA showed an effect of altering the rules of the SSG on the dependent variables [Pillai's Trace = 0.26; F_(9,240)_ = 2,53; *p* = 0.009; *η*^2^*p* = 0.087, moderate effect]. There were significant differences in the length (F = 5.91; *p* = 0.001; *η*^2^*p* = 0.180, moderate effect), width (F = 3.37; *p* = 0.022; *η*^2^*p* = 0.112, moderate effect), and LpWratio (F = 2.81; *p* = 0.045; *η*^2^*p* = 0.09, moderate effect) when comparing the R1 (higher) and FR (lower) protocols. For the distance between the centroids, Friedman's test did not report significant differences (F = 1.92; *p* = 0.145; *η*^2^*p* = 0.149, moderate effect). In summary, players tended to stretch the pitch occupation when playing under the influence of the explicit rule. Figure 3 shows the results.

**Figure 4:**
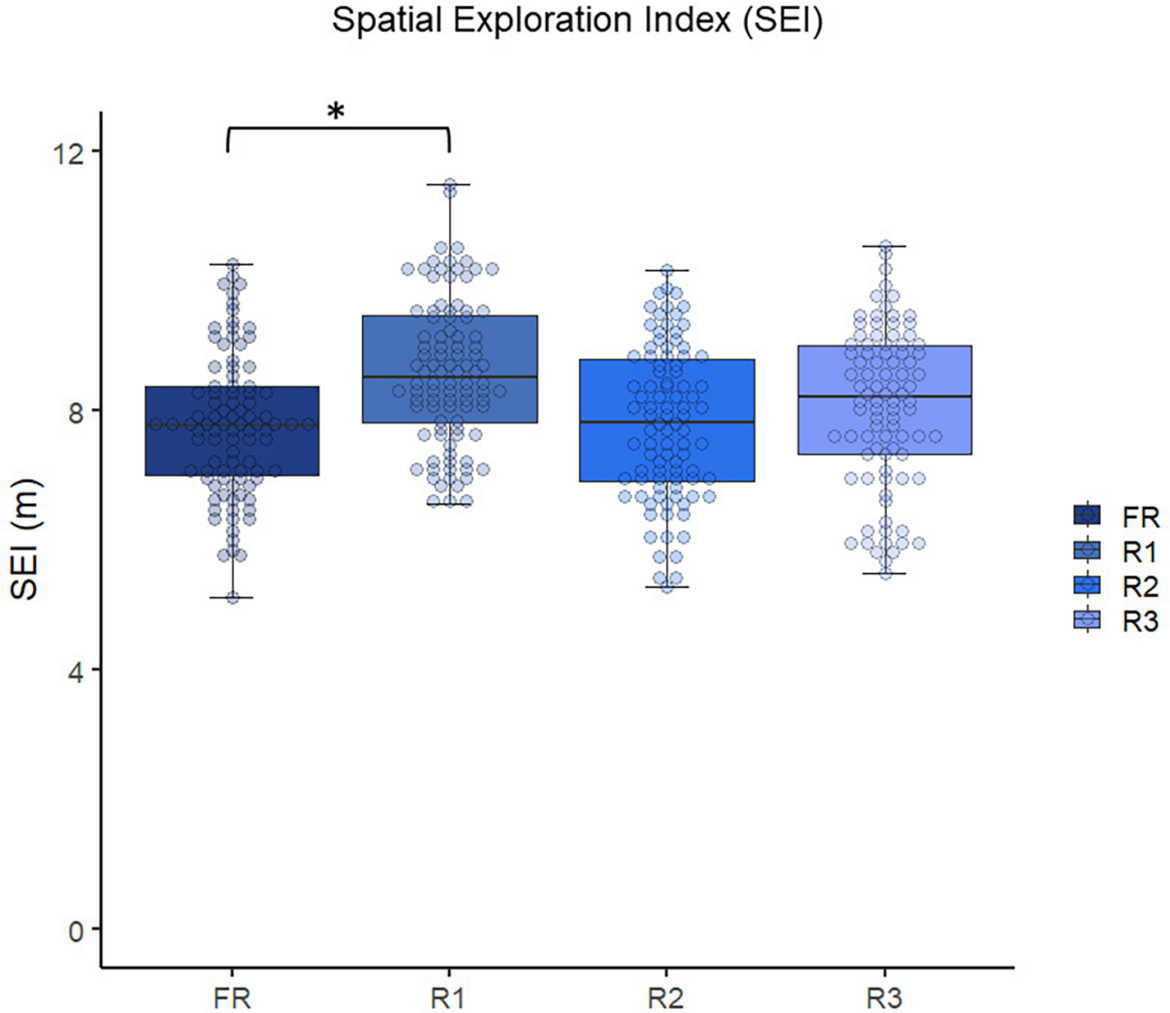
Comparison of the SEI between the four experimental conditions. *significant differences.

When analyzing the SEI, the ANOVA indicated a main effect of the experimental condition on the observed values [F_(3,81)_ = 12.92; *p* < 0.001; *η*^2^*p* = 0.135, moderate effect]. The highest SEI was observed in the R1 condition (8.6 ± 1.1), significantly higher than all the other experimental conditions [R2 = 7.8 (1.1); R3 = 8.1 (1.1); FR = 7.7 (1.0)]. This means that players moved to different regions of the pitch more constantly in the explicit rule condition. Figure 4 shows the results regarding the SEI.

**Figure 5:**
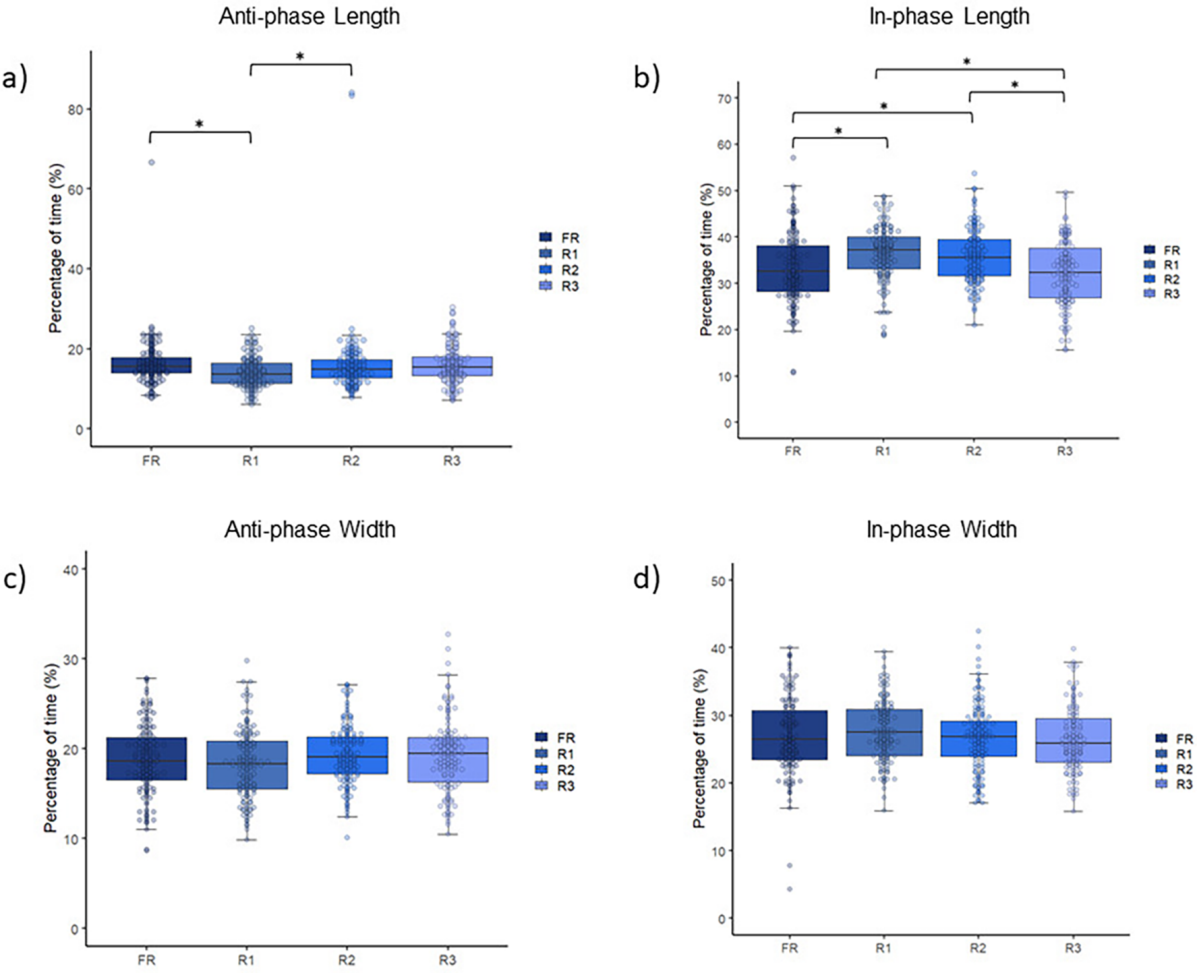
Comparison of the **(A)** in-phase length, **(B)** anti-phase length, **(C)** in-phase width, and **(D)** anti-phase width between the four experimental conditions. *significant differences.

Finally, the MANOVA showed a significant effect of changing the rule on the interpersonal coordination tendencies in both in-phase and anti-phase [Pillai's Trace = 0.09; F_(12,1626)_ = 4.51; *p* < 0.001; *η*^2^*p* = 0.03; no effect]. For longitudinal displacements, the ANOVA showed an effect of protocol on anti-phase interpersonal coordination (F = 4.34; *p* = 0.005; *η*^2^*p* = 0.02, no effect), lower in the R1 condition than in the R2 and FR conditions, and for in-phase interpersonal coordination F = 13.05; *p* < 0.001; *η*^2^*p* = 0.07, moderate effect), higher in the protocols R1 and R2 than in the protocols R3 and FP. No differences were observed in the in-width displacements. Figure 5 shows the data regarding this analysis. These results indicate that the presence of rules allowed players to better coordinate their movements during the game.

## 4 Discussion

This study aimed to compare the tactical behavior and performance of U-20 soccer players during small-sided games (SSG) with implicit and explicit informational constraints. Four distinct experimental conditions were examined, namely, a free rule (FR), an explicit rule (R1), an implicit rule (R2), and a combined rule (R3). The main results indicate that players achieved a higher percentage of in-phase coordination tendency in the explicit rule condition, which confirms the hypothesis. Also, it has been shown that summing up implicit and explicit rules can hinder players' performance. Finally, the explicit condition leads players to occupy the pitch in a stretched way, indicated by higher length and width values. The study's innovation lies in exploring how implicit and explicit informational constraints affect players' performance in high-complexity tactical scenarios. The anticipated outcome was a superior tactical performance under explicit conditions, substantiated by the findings, although the effect sizes are small.

In the current study, players showed higher in-phase synchronization (in-length) values in the explicit instruction condition than in the FR. According to Raab (13), explicit learning is more advantageous than implicit when complex tasks are learned. For example, in soccer, the high-press defensive action should be considered a highly complex task as it requires players to effectively and timely coordinate their actions to achieve the collective goal (20), which explains why players showed higher in-phase coordination in the explicit rule condition. Lola et al. (14) in a task involving novices in a badminton decision-making task. Besides, Batista et al. (62) observed that explicit defensive technical instruction increased the ball recovery in inner zones compared to a non-instruction condition in semi-professional soccer players. Raab (13) argues that some explicit learning, guided by “if-then” rules (such as “if you regain the ball further on the pitch, you will score an additional point”), is required for players to learn how to map appropriate decisions during the tasks. Therefore, as explicit learning is required during specific decision-making in sports when perceptual-cognitive requirements are high, the tactical performance during the defensive high-press benefits from providing players with explicit instructions—experimental condition R1 - which explains the current results.

Interestingly, the protocol combining the two rules did not induce similar differences as observed in the explicit instruction condition. Although players received the same information, they adopted a game pattern close to the control situation (without additional rules). Even if informational constraints can modify players' actions (63), these constraints can emphasize some specific affordance landscapes (64), and exaggerating the information provided might lead players to neglect them and adopt a free-play pattern. In line with this, Machado et al. (65) observed a low attacking pattern variability when more rules were added to the game. Therefore, from a pedagogical point of view, coaches should cautiously increase the number of rules in a given game, as providing players with excessive information can reduce their ability to explore affordances and promote few self-organization and adaptation actions. This is particularly important considering the current study sample comprised high-level U-20 players from elite youth academies. Notwithstanding, the experience and accumulated training hours did not reduce the impairment caused by the excessive number of rules provided in the R3 condition, which could indicate a more deleterious effect in younger groups.

Besides the abovementioned rationale, it is worth pointing out the role of the players' synchronization measurement as a performance indicator in soccer (38). Sampaio and Maçãs (66) argued that interpersonal coordinated movements are fundamental in improving players' skills. This coordination seems to be also related to players' experience, as Figueira et al. (67) showed that older players present better intra-team synchronization. However, it seems essential to consider the coordination regarding the pitch's axes (longitudinal and lateral). At this point, Duarte et al. (68) verified that professional soccer players are keener to coordinate their behavior in-depth than in width due to the attraction caused by the goals. A similar result was also observed in the current study. Therefore, future studies are encouraged to expand the link between coordination and performance, looking primarily at the in-depth coordination patterns.

In the current study, players' spatial occupation was also measured. At this point, higher SEI, width, length, and LpWratio were observed in the R1 condition compared to the FR. Therefore, it can be assumed that the explicit rule led to a more spread and variable tactical positioning on the pitch. Previous studies showed a higher SEI when the players were less familiar with the task (69–71). For example, higher values of SEI were observed in the progression-to-the-target SSG than in the regular format (71). Also, Clemente (69) showed higher SEI values in the half-size condition compared to the full-size. Therefore, it could be assumed that the high-press defensive strategy, which was more prominently emphasized in the explicit-rule condition, generated a less familiar tactical scenario, which required a higher tactical exploration of the players, which explains the current findings. Another possible explanation is related to the area to cover during the game. Gonçalves et al. (72) showed that higher SEI values are associated with larger covered areas during the games. Therefore, pressing the opponent high, emphasizing more clearly the explicit rule condition, enlarged the areas to cover, and increased the SEI values. Therefore, the main explanation for the higher SEI values found in the current study in the R1 condition might rely not on the nature of the rule (explicit vs. implicit) but on the intended behavior (defensive high-press).

Besides, the R1 condition elicited higher values of length, width, and LpWratio, which indicates players' tendencies to enlarge the team's shape during this condition. The previously discussed higher SEI might explain the larger collective spatial occupation of the players. At this point, Barnabé et al. (73) showed that older and more experienced players exhibited a higher dispersion and expanded positioning during the attack. As the current study analyzed the tactical behavior of highly trained U-20 athletes, it is assumed they are keener to enlarge the team shape when required (e.g., during a defensive high-press action from the opposing team), which aligns with the current findings. Also, Praça et al. (71) suggested that off-the-ball players should move to create passing opportunities far from the ball to allow the team to progress toward the opposing goal, which could increase the spatial occupation. In the present study, due to the tougher defensive press observed in the R1 condition, offensive players might similarly enhance their off-the-ball movements by exploring more spaces (higher SEI) and enlarging the team shape (higher length and width), which explains the current findings. Although high-press might rely on the compactness of the defending team (74), the length might be higher due to the impossibility of keeping compactness in the offensive midfield (due to the offside rule). Together, the higher length during high-press, the need to increase the team shape from the offensive team, and the players' ability to adjust their behavior to these new contextual features explain the current findings.

For the distance between the teams' centroids, there were no significant differences between the experimental conditions. This result is in line with a previous one (75) that showed no differences between the teams' centroids' distances in different game moments, which suggests a low sensibility of this variable to detect changes concerning game strategies (such as the high-pressing). On the other hand, Folgado et al. (20) found a higher team's centroids distance in the 3 vs. 3 game than in the 4 vs. 4 in U-13 soccer players. However, the authors did not report using the offside rule, which might explain this divergence. Specifically, players are encouraged to move deep on the field without the offside rule, increasing the length (52). Consequently, players from the defending team are encouraged to protect their own goal instead of pressing high (76), which might have caused the higher centroid distance observed. Also, Frencken et al. (77) compared the teams' centroid positions and verified a strong association between the two teams in the longitudinal axis (length of the pitch) and that smaller pitches resulted in lower teams' centroid distances. Therefore, the tendency of coupling in-length displacements and the reduced available pitch in the current study might have reduced the possibility of the teams adapting their position—concerning the position of the team's centroid—under different experimental conditions. Notwithstanding, future studies in larger pitches are encouraged to test such an assumption.

The present study has some limitations that should be considered carefully. One is that game phases were not analyzed separately, as suggested in the literature (31, 78). This may have affected the results on positional data during game phases as the differences between defense and offense positioning strategies (e.g., high-press vs. low-block) were not captured. This also is likely to increase the variability of the data, which might explain the current small effect size values for some variables. Therefore, it is suggested that future studies develop methods to split position data into game phases. For this reason, future studies should test the link between instructional constraints and defensive high-press in larger pitches. Also, it has been shown that machine learning and artificial intelligence techniques might be useful in identifying key performance indicators in soccer (79–81). Therefore, it would be useful to integrate the current research problem into a multidimensional performance analysis to identify which performance components are strongly influenced by explicit/implicit instructions. Finally, the findings may not reflect the chronic effects of positional play; thus, longitudinal studies are encouraged.

From a practical standpoint, coaches should consider that the way information is provided to the players acutely impacts their performance in game-based tasks. When training complex tactical content, providing explicit information can enhance tactical performance, even if only in the short term. This approach is particularly useful in pre-match training sessions, where acute strategic adjustments are often emphasized. However, overloading players with additional information and combining implicit and explicit instructions may diminish performance and impede tactical learning. For this reason, choosing the right information seems more important than providing extensive, cumulative instructions.

## 5 Conclusion

Providing the players with explicit instructions on complex tactical concepts significantly increased their performance. From a practical point of view, this means that coaches should consider incorporating explicit tactical instructions in their training regimens to enhance player adaptability and tactical execution. On the other hand, summing up instructional constraints can impair players' self-adaptation processes during the game. Therefore, coaches should carefully select information to be given to the athletes, as the “more-is-better” approach has been denied by the current study. Furthermore, the explicit instruction condition was the only one showing regular differences from the control (no instructions) condition. Finally, the tactical performance was higher under the explicit instruction condition and characterized by a game with larger pitch occupation (higher length and width) and spatial exploration (SEI).

## Data Availability

The raw data supporting the conclusions of this article will be made available by the authors, without undue reservation.
